# Photosynthetic function analysis under rhizosphere anaerobic conditions in early-stage cassava

**DOI:** 10.1007/s11120-025-01163-4

**Published:** 2025-07-30

**Authors:** Lado Aquilino, Kirana Luthfia Nayatami, Alex Tamu, Ibrahim Soe, Jun-Ichi Sakagami

**Affiliations:** 1 The United Graduate School of Agricultural Sciences, Kagoshima University, Kagoshima City, Japan; 2 Graduate School of Agriculture, Forestry and Fisheries, Kagoshima University, Kagoshima City, Japan; 3 Faculty of Agriculture, Kagoshima University, Kagoshima City, Japan; 4 Directorate of Research and Training, Ministry of Agriculture and Food Security, Juba City, Republic of South Sudan; 5 Sierra Leone Agricultural Research Institute, Free Town, Sierra Leone

**Keywords:** Waterlogging, Cassava, Chlorophyll fluorescence, Leaf gas exchange, Photosynthesis, SPAD

## Abstract

To enhance land use efficiency and meet rising cassava demand, cultivation is expected to expand into unsuitable lowland areas. This trend highlights the need for waterlogging-tolerant cassava genotypes. However, research on cassava survival mechanisms under waterlogged conditions through photosynthetic functions remains limited. This study investigated the physiological responses of cassava to waterlogging stress. It focused on photosynthesis, stomatal conductance, soil plant analysis development (SPAD), and chlorophyll fluorescence (Fv/Fm) to determine chlorophyll degradation and its effect on photoreceptors. Cassava was subjected to waterlogging by maintaining water-filled buckets throughout the treatment. Variables were measured periodically at intervals of 0, 3, 6, 9, 12, and 15 days after treatment (DAT). Results showed a reduction of net photosynthetic rate (A) by 82.6%, resulting from a 96.7% reduction in stomatal conductance (gs) and 21% in transpiration rate (E). A, gs, and E in three-month-old cassava varied and declined with increasing waterlogging duration, while SPAD value showed no significant differences compared to the control across all measurement dates. Fv/Fm showed a significant decrease at 3DAT followed by recovery, likely due to light de-excitation rather than chlorophyll degradation, as SPAD value remained unchanged, indicating no chlorophyll breakdown or photoreceptor damage in three-month-old cassava under waterlogging conditions. The study concluded that cassava exhibits a functional stay-green type of SPAD, and photosynthetic nitrogen use efficiency, along with stomatal and nonstomatal limitations that regulate photosynthesis under waterlogged conditions. Study provides insights into how cassava cope with waterlogging and guide breeding or agronomic strategies to improve their resilience in waterlogged environments.

## Introduction

With the global population rising, food demand is projected to increase by up to 110% over the next 30 to 35 years (De Souza et al. 2017), while the population of sub-Saharan Africa alone is expected to increase by over 120%. In this region, cassava (*Manihot esculenta*) is the second-most important source of calories and accounts for approximately 30% of the daily calorie requirements per person. Despite its importance, the average cassava yield in Africa has shown little improvement since 1961 (De Souza et al. 2017). Photosynthesis performance during early vegetative growth is a key physiological trait influencing crop yield, yet studies on photosynthesis in cassava remain limited. Waterlogging is widely recognized as an abiotic stressor that limits photosynthesis and growth in most plant species (Maai 2024). The earliest response of plant species to waterlogging is stomatal closure, which limits gas exchange and decreases A and E in many plant species (Ahmed et al. 2002).

Waterlogging has become a major abiotic stressor for crops due to climate change, causing stunted growth and decreased productivity (Chen et al. 2022). Water excess is a key environmental factor affecting plant growth and development; prolonged flooding limits soil oxygen availability, leading to hypoxia (low oxygen levels) or anoxia (complete oxygen deprivation) Taiz et al. (2017). Underground plant organs, such as roots and rhizomes, are the first to be exposed to soil waterlogging, and both organs preferentially function under aerobic conditions. According to Perl-Treves and Perl (2001), oxygen atom is the most abundant element in the Earth’s crust, and its presence in molecular form (O_2_) in the biosphere is essential for sustaining aerobic life. Oxygenase enzymes are the main oxygen fixers in organic molecules (Perl-Treves and Perl 2001). Under waterlogged conditions, oxygen scarcity impairs enzyme activity and disrupts normal physiological processes. Thus, waterlogging limits stomatal and hydraulic conductance, leading to reduced photosynthesis and respiration, and ultimately suppressing plant growth (Vartapetian et al. 2003). With growing recognition of cassava’s importance, its cultivation area is expanding, including into unsuitable lowland regions. As a result, cassava is increasingly exposed to excess water during the rainy season, placing it at risk of soil waterlogging stress- the situation of excess water in the root zone where soil pores that normally would be gas-filled become water-filled (Voesenek and Colmer 2009). However, research on the waterlogging tolerance mechanism of this species remain scarce. Photosynthesis is a fundamental process in crop production and a key factor influencing growth and yield. As such, investigating the physiological responses of cassava to waterlogging stress particularly photosynthetic responses is essential, as measured by leaf chlorophyll content and chlorophyll fluorescence to assess chlorophyll degradation and its impact on photoreceptors (Chen et al. 2022).

Previous literatures report that, cassava exhibit two distinct physiological and morphological responses to waterlogging stress (Dethvongsa et al. 2021). First, cassava exhibits early leaf yellowing, beginning with older leaves, followed by shedding of the yellowed leaves. Second, whole-plant withering occurs, with the lower leaves drying and falling off, leaving only a few young leaves at the top. At the vegetative stage, cassava shows recovery in aboveground parts after 12 days of waterlogging, but not in storage roots, highlighting the potential of using the leaf retention ratio as a screening tool for waterlogging-tolerant germplasm or breeding lines (Kongsil and Nakasathien 2021). Wang et al. (2021) reported that hypoxia developed in cassava roots, with oxygen concentration sharply decreased from the outer layers toward the inner tissues. They also found that the expressions of the MePFK03, MePFPA1, and MePFPB1 genes declined in response to reduced oxygen levels in the root. Furthermore, high-throughput sequencing was used to characterize transcriptome responses in the roots and leaves of partially submerged cassava plants, resulting in the identification of 15,902 differentially expressed transcripts (Chen et al. 2022). Kyoto Encyclopedia of Genes and Genomes enrichment analysis comparing leaf samples under non-waterlogged conditions with those under waterlogging and root samples under non-waterlogged conditions with those subjected to waterlogging reveals that waterlogging stress mainly represses photosynthetic reactions in the leaves and improves energy savings in the roots. Amino acid metabolism was also substantially altered in both tissues, and induction of a nitrate-producing pathway may help maintain ATP levels (Chen et al. 2022). Chen et al. (2022) observed complex interactions between energy production and the antioxidant enzyme system, suggesting that both systems play important roles in the waterlogging response. According to Alves et al. (2024), cassava plants suffer damage and leaf death under rhizosphere hypoxia. In addition, chlorophyll fluorescence decreases rapidly immediately after treatment, with a greater reduction than in photosynthetic rate, indicating that O₂ deficiency in the rhizosphere also impacts aboveground tissue. However, the mechanism underlying this effect remains unknown. Therefore, the objective of this study was to investigate the main factors underlying reduction of the physiological responses particularly photosynthetic responses of cassava under waterlogging conditions. Using leaf gas exchange and chlorophyll fluorescence measurements, we tested the hypothesis that stomatal and nonstomatal limitations of photosynthesis are the main factors reducing the net photosynthetic rate in cassava under waterlogging conditions. Understanding these physiological adaptations will provide valuable insights into how cassava cope with waterlogging stress and guide breeding or agronomic strategies to improve their resilience in waterlogged environments.

## Materials and methods

### Plant materials

The Tokunoshima Yellow 2 cassava genotype (*Manihot esculenta* Crantz), the most common cassava variety cultivated in Tokunoshima in Japan, was used in this study. Because cassava plants cannot survive outdoors during the winter in Kagoshima, cassava cuttings (planting materials) for this experiment were prepared from cassava stems harvested in December 2023. Each stem was pruned to a 1.5-m length, tied into bundles, and preserved by placing them into plastic containers filled with river sand. The containers were then covered with clear plastic sheets to maintain temperature and humidity and stored in a plastic greenhouse.

### Experimental design

This experiment was conducted in a greenhouse at Kagoshima University, Faculty of Agriculture. When preparing the cassava cuttings, germinated and rooted parts were removed, and stems without roots and leaves were cut into 20-cm length for planting. The prepared 20-cm cassava cuttings were first soaked in Hyponica solution for 24 h to promote leaf growth and rooting and then planted into plastic buckets (20 cm wide × 60 cm long × 20 cm deep) filled with loam and sand soil in a 4:1 ratio. On September 3, 2024, when all plants had attained the three-month growth stage, six cassava seedlings per bucket were selected based on the SPAD values measured for each seedling. Buckets containing six relatively uniform cassava seedlings were then chosen for the experiment. Six buckets, each containing six cassava seedlings, were selected and transferred into two big buckets, three per big bucket set for the experiment: one big bucket for waterlogging (WL) treatment and the other one for well-watered (WW) treatment (control). The waterlogging treatment was initiated on September 3, 2024, by dipping the small planting buckets in a big bucket filled with water. Three replications were maintained for each treatment. The water level in the waterlogged bucket was maintained 3–4 cm above the cassava stem and soil surface on the cassava planting buckets.

### Data collection and measurements

Data was collected at 0, 3, 6, 9, 12, and 15 DAT. Three replications within two buckets per respective treatment and sampling point were used for data collection. The parameters measured comprised leaf gas exchange parameters—A, E, gs, and intercellular CO_2_ concentration (Ci)—Fv/Fm in dark-adapted leaves, and SPAD. The photosynthetic water use efficiency (PWUE) was calculated by dividing A by E at the same CO_2_ concentration. Average daily air temperature and relative humidity were measured using a sensor equipped with a data logger (RTR-503, T&D Corporation, Japan), and soil temperature and soil moisture content were measured using a sensor (5TE, METER Group Inc.; USA). The average daily light intensity in the glasshouse was measured using sensors equipped with a data logger.

#### SPAD measurement

On the same day as the photosynthesis measurements, the SPAD values for three plants in each treatment were recorded using a SPAD meter (SPAD502; Konica Minolta). According to Uddling et al. (2007), the SPAD meter produces SPAD values, typically ranging from 0.0 to 50.0, which are proportional to the amount of chlorophyll present in the leaf. SPAD values were determined from three successive leaf positions in each plant. The third fully expanded leaf of each plant was chosen for SPAD value determination. SPAD measurements were taken from the same leaves used for chlorophyll fluorescence assessment.

#### Fv/Fm measurement

Fv/Fm was measured under dark adaptive conditions on fully developed leaves using AquaPen (AP 100-P, photon systems Instruments, Czech Republic). The maximum yield of PSII photochemistry, calculated as variable fluorescence (Fv) divided by maximum fluorescence (Fm), was obtained by emitting actinic light through the quantum yield menu. Chlorophyll fluorescence was measured on the same day as the photosynthesis measurement. However, the measurement was performed at night in the absence of light. Three replicates per treatment were used for Fv/Fm data collection.

#### Leaf gas exchange parameters

Leaf gas exchange parameters (A, E, gs, and Ci) of the central foliole of the youngest fully expanded leaf on one plant/replication (*n* = 1) were measured on the 3-month-old plants using a portable gas exchange system integrated with a leaf curette, including a modulated chlorophyll fluorometer and light source (LI- 6400XT In., Lincoln, Neb., USA) equipped with the standard 2 × 3 cm leaf chamber (6400-08 clear chamber bottom). The leaf was acclimated to a saturating light intensity of 1500 µmol m^− 2^ s^− 1^and CO_2_ concentration of 400 µ mol mol^− 1^ inside the cuvette. After steady-states for A and gs were obtained, measurements were recorded. The block temperature was set to 28 °C, and VPD inside the cuvette was maintained at the air flow rate at 500 µ mol s^− 1^. In addition, gs and operating Ci were obtained from the data points collected at 400 µ mol^− 1^ (CO_2_). PWUE was calculated by dividing A by E at the same CO_2_ concentration. Leaf gas exchange parameters were measured periodically from 9 am to 12 pm.

### Statistical analysis

Data were analyzed in IBM SPSS statistics (Version 27.0.1.0) using univariate analysis of variance to determine the single and interaction effects of the treatment day interval (treatment duration) (D0–D15) and water treatment (WW and WL). The mean treatments from replications were compared using a Turkey’s HSD test at 0.05% level (*p* < 0.05). The mean values from WL treatments were compared with those of WW treatments using t-test. Pearson correlation matrix analysis was performed to test for correlations between parameters, in which Pearson’s correlation coefficients between the parameters were calculated.

## Results

### Growth environment, air temperature, relative humidity, soil temperature, soil moisture content, light intensity, and bulk EC

**Fig. 1:**
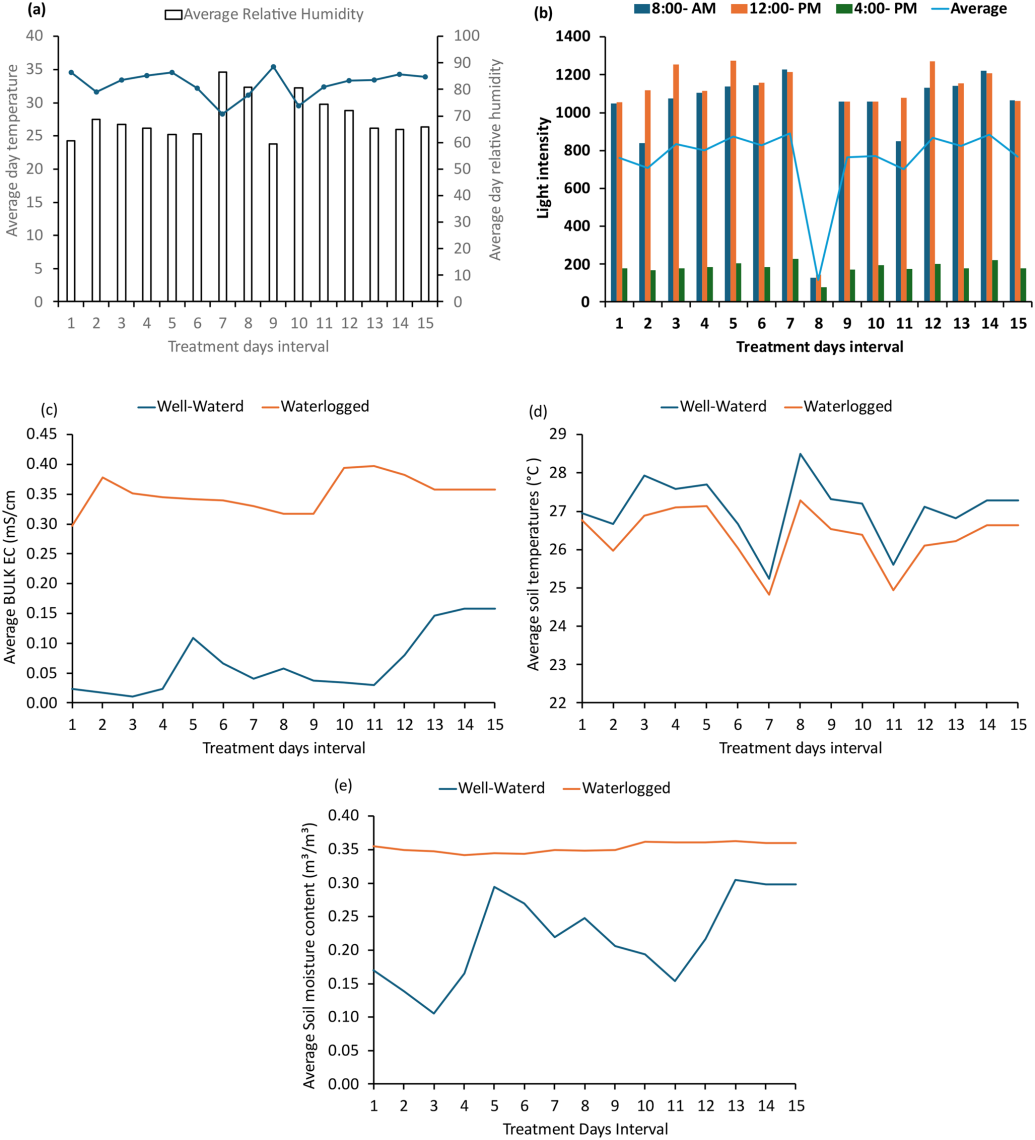
Average day temperature and average day relative humidity (**a**), light intensity (**b**), Average Bulk EC (**c**), Average soil temperature (**d**) and Average soil moisture content (**e**) during experimental duration

Cassava growth and production are favored by average annual temperatures above 20 °C and air humidity between 60% and 70%. During the experimental period, the temperature and relative humidity in the glasshouse remained within this ideal range, supporting optimal cassava growth. The daily mean temperature in the greenhouse during the experimental period was 33 °C, ranging from 29.5 °C to 35.4 °C. The mean relative humidity was 69.17%, with values ranging from 59.5 to 86.4% (Fig. 1a). The obtained mean light intensities were 8:00 (1015.40), 12:00 (1081.40), and 4:00 (181.33) (Fig. 1b). The volumetric water content was 0.11–0.30 m³/m³ (mean: 0.22 m³/m³) for the WW treatments and 0.35–0.36 m³/m³ (mean: 0.35 m³/m³) for the WL treatments (Fig. 1c). The soil temperature was 25.24–28.50 °C (mean: 26.94 °C) for WW and 24.83–27.29 °C (mean: 26.31 °C) for WL (Fig. 1e) throughout the experimental duration. The bulk EC values were 0.01–0.06 ms/cm (mean: 0.06 ms/cm) for WW and 0.23–0.40 ms/cm (mean: 0.34 ms/cm) for WL (Fig. 1d) throughout the experimental duration.

### Effect of waterlogging on leaf gas exchange parameters

**Fig. 2:**
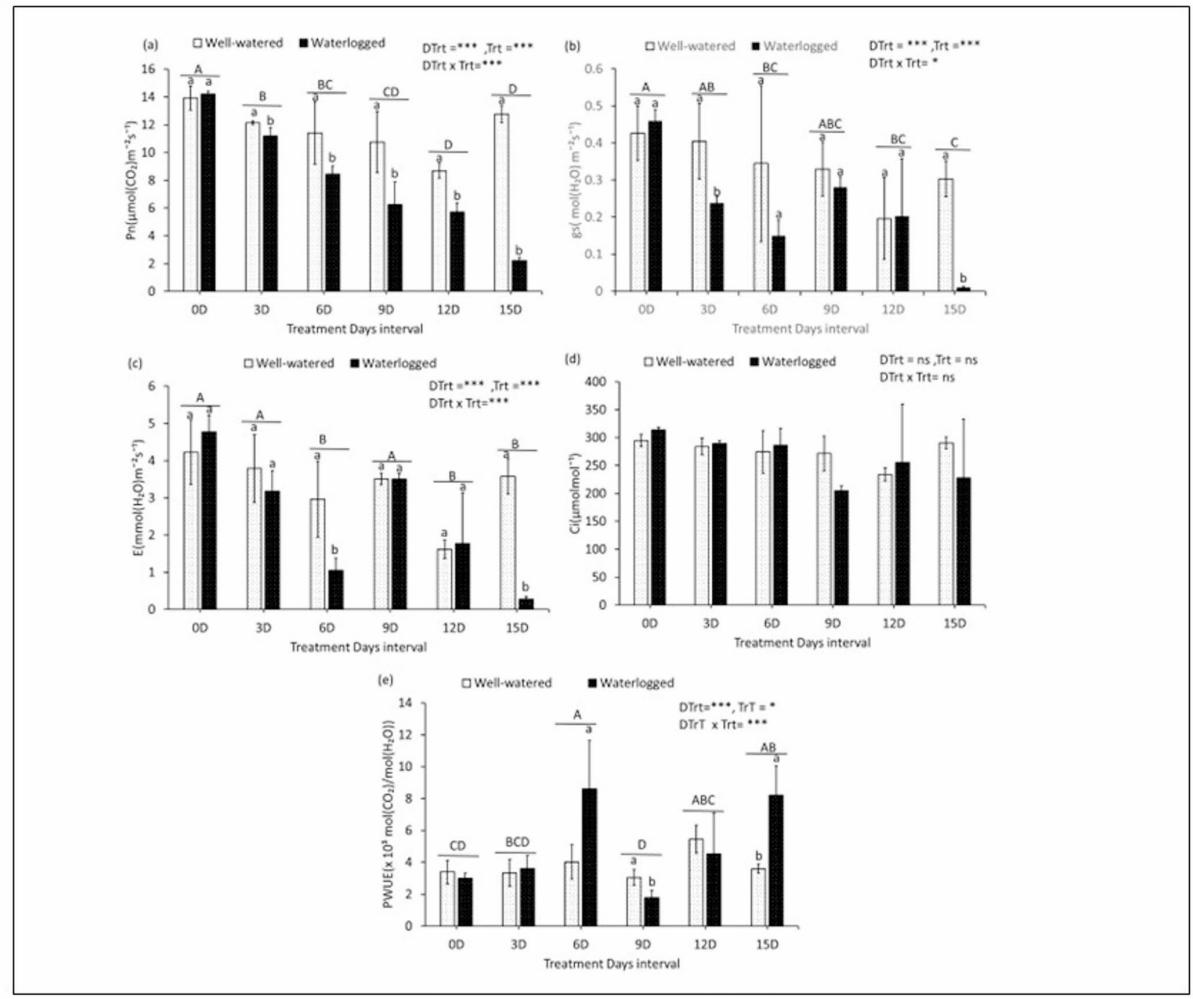
Effect of waterlogging on gas exchange parameters (A (**a**), gs (**b**), E (**c**), Ci (**d**), and PWUE (**e**)) in Cassava plants. Values are the means ± standard deviation (*n* = 3)

Figure 2a–e shows the effect of waterlogging on leaf gas exchange parameters—A, E, gs, and Ci—and the calculated PWUE of the evaluated cultivar grown under WW and WL treatments.

WL cassava plants showed significantly lower mean A than WW controls by 7.9% (3DAT), 26.0% (6DAT and 9DAT), 48.8% (*P* < 0.05) (12DAT), and 82.6% (*P* < 0.001) (15DAT). While WL exhibited 1.9% higher A on 0DAT, no treatment differences persisted beyond day 15 (Fig. 2a). WL plants showed significant reductions in gs—96.7% (*P* < 0.01) (15DAT) and 41.1% (3DAT)—compared to the WW controls. Nonsignificant decreases of 56.6% (6DAT), 33.7% (9DAT), and 9.2% (12DAT) were also observed. However, gs was 7.2% higher in WL than WW plants at 0DAT (Fig. 2b). WL plants had lower E variations than WW controls—significant values at 64.5% (6DAT) and 92.2% (15DAT) and nonsignificant values at 16.1% (3DAT), 32.1% (9DAT), and 30.5% (12DAT)—but higher values on 0DAT (11.4%) (Fig. 2c). WL plants showed nonsignificant increases in Ci relative to WW controls—6.1%, 1.9%, 4.1%, and 0.8% on days 0DAT, 3DAT, 6DAT, and 12DAT, respectively—and nonsignificant decreases in Ci, 11.5% (9DAT) and 21.4% (15DAT) (Fig. 2d). Furthermore, PWUE in WL plants showed mixed responses compared to WW controls. While PWUE was non-significantly reduced by 11.7% (0DAT), 41.4% (9DAT), and 16.8% (12DAT), it increased by 7.6% (3DAT), 53.3% (6DAT), and 56.2% (15DAT) (Fig. 2e). Contrary, Fig. 2a, b & c also show decreasing trends of leaf gas exchange parameters A, gs and E even in the well-watered plants as the treatment duration increased.

### Effect of waterlogging on SPAD and fv/fm

**Fig. 3:**
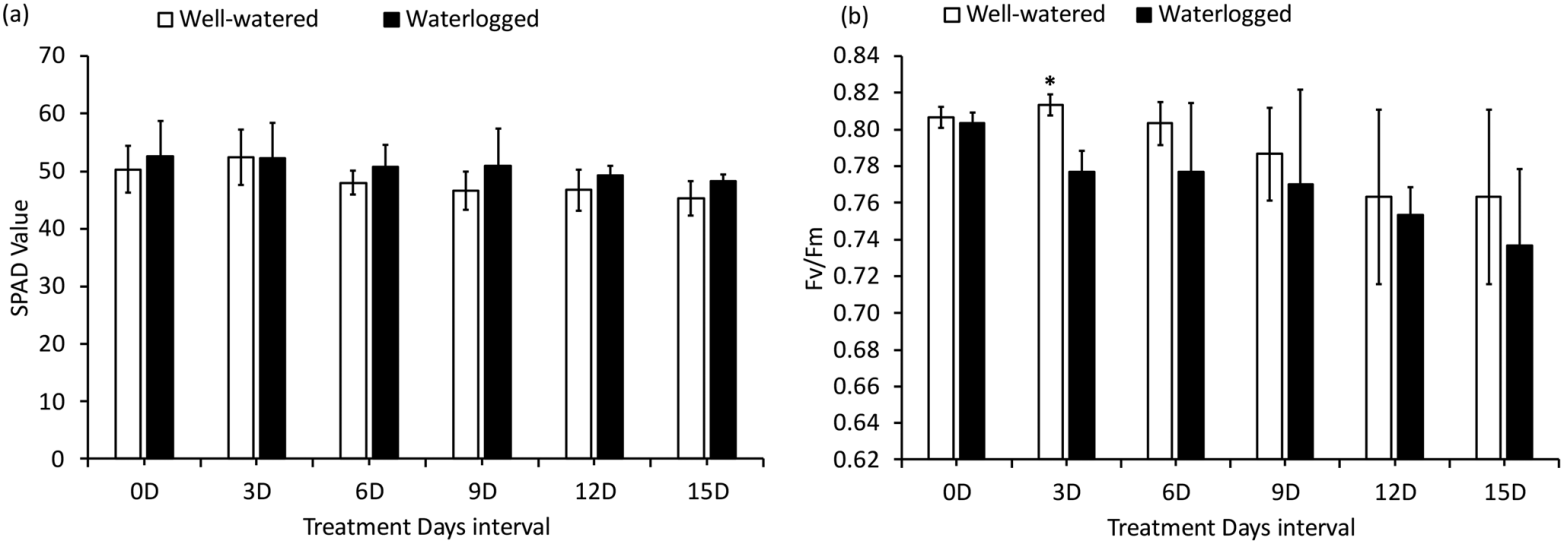
Effect of waterlogging on SPAD value (**a**) and chlorophyll fluorescence (Fv/Fm) (**b**). Values are the means ± standard deviation (*n* = 3)

SPAD values, which indicates the relative chlorophyll content, did not significantly differ among the WL and WW treatments for each paired measurement. However, the SPAD values increased over time across the measurement periods as plant growth progressed (Fig. 3a). Meanwhile Fig. 3b shows the results based on Fv/Fm trait for the cultivar grown under WL and WW. Fv/Fm trait only differed significantly (*P* > 0.05) among the WL and WW treatment on 3DAT. However, the Fv/Fm trait did not differ thereafter across the measurement periods (Fig. 3b).

### Pearson correlation matrix of leaf gas exchange and photosynthetic parameters

**Fig. 4:**
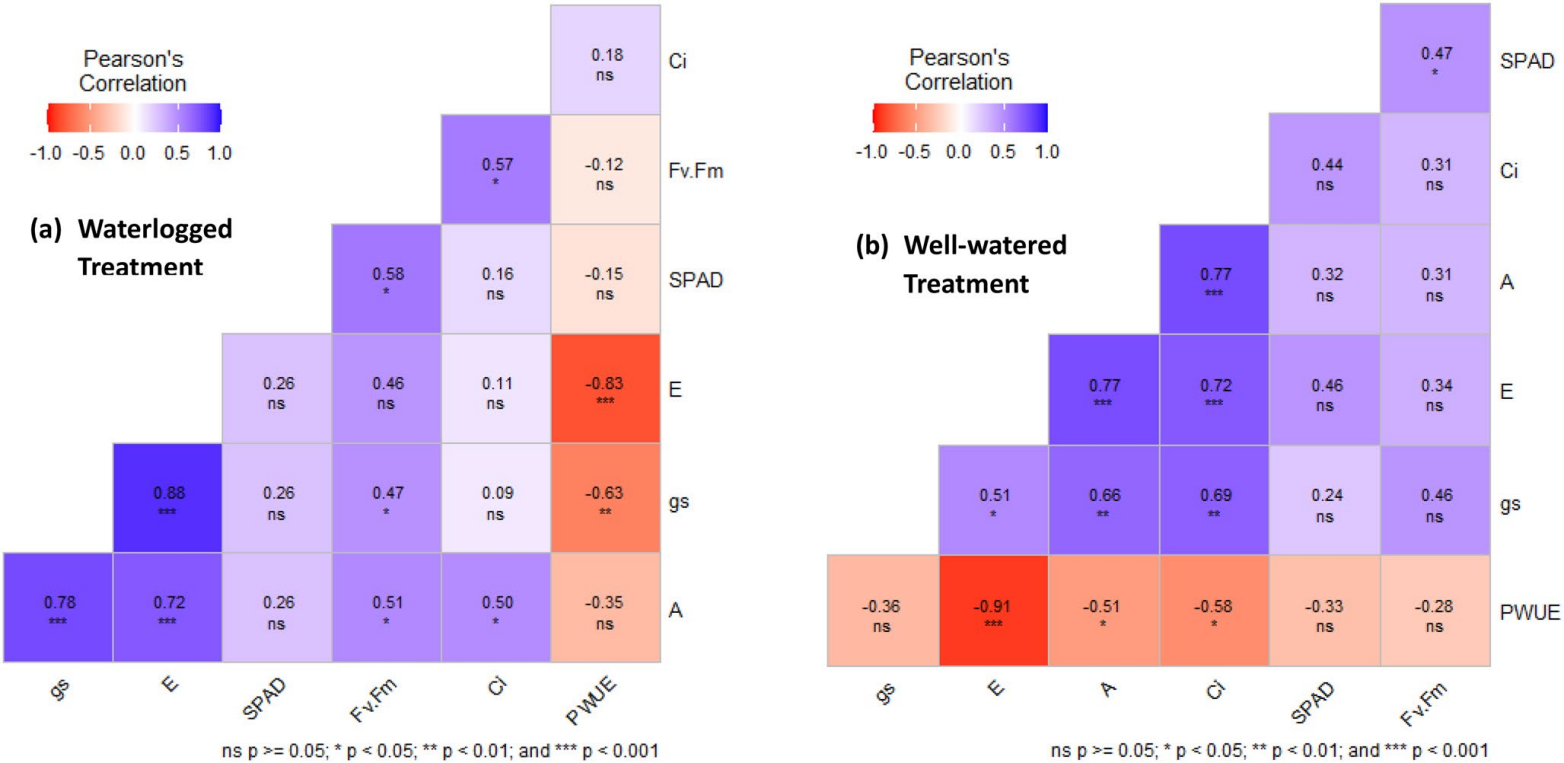
Relationship between leaf gas exchange parameters and related photosynthetic parameters (**a**) Waterlogged treatment and (**b**) Well-watered Treatment

Figure 4a shows trend of Pearson correlation matrix performance of waterlogged treatment related traits. Where significant positive correlations between A and gs (*P* < 0.001), E (*P* < 0.001), Ci (*P* < 0.05) and Fv/Fm (*P* < 0.05) were observed. However, A was negatively non-significant correlated with SPAD value and PWUE. In addition, gs exhibited a significant positive correlation with E (*P* < 0.001), Fv/Fm (*P* < 0.05) and non-significantly correlated with Ci, SPAD values and negatively significantly correlated with PWUE. Meanwhile Fig. 4b shows trend of Pearson correlation matrix performance of well-watered treatment related traits. Where A shows significantly positive correlation with gs (*P* < 0.01), E (*P* < 0.001), Ci (*P* < 0.001) and non-significantly correlated with SPAD value and Fv/Fm. Furthermore, gs exhibited a significant positive correlation with E (*P* < 0.05), Ci (*P* < 0.01) and non-significantly correlated with SPAD values and Fv/Fm as well as negatively non-significantly correlated with PWUE.

## Discussion

This study investigated the physiological responses of cassava plants to waterlogging stress by analyzing key photosynthetic parameters, including stomatal conductance, leaf chlorophyll content, and Fv/Fm—aiming to determine chlorophyll degradation and its impact on photoreceptors.​ Results of SPAD values, which indicates the relative chlorophyll content in this study was found to be non-significantly different between treatments or over treatment duration, likely due to the functional stay-green type of SPAD value (Fig. 3a): cassava plants can maintain higher PNUE under nitrogen-deficient conditions, such as those induced by waterlogging. This was in agreement with Cruz et al. (2003) who reported that cassava can acclimatize to nitrogen starvation as it has evolved under conditions of low nitrogen availability. Transcriptomic profiling studies have identified candidate molecular responses to waterlogging in cassava. For instance, notable alterations in amino acid metabolism were observed in cassava roots and leaves under waterlogged conditions; a nitrate-producing pathway was induced, potentially aiding in ATP level maintenance during hypoxic stress (Chen et al. 2022). Furthermore, Maai (2024) reported that 33 and 56 days of long-term waterlogging increased SPAD, resulting in increased A values. Ikezawa et al. (2015) also demonstrated an increase in SPAD under 94- and 125-day waterlogging in Eddo. These studies confirm that waterlogging effects are plant species-specific and treatment duration specific. The adverse effects of waterlogging are also growth stage-specific (Araki 2006; Baranwal and Singh 2002; Ren et al. 2014). Soil chemical property changes may have affected SPAD values, potentially explaining the cassava SPAD response observed in this study. Consequently, 3-month-old cassava seedlings subjected to 15-day waterlogging stress utilized PNUE as a survival mechanism, which explains the absence of chlorophyll degradation observed in the current study. The results of SPAD Values also indicate that the decreased light intensity on day 8th (Fig. 1b) which was caused by rain cloudy day has not affected the parameters of photosynthesis. This could be because, immediately the following day after the release of that temporary induced-light stress, cassava might have recovers rapidly and increase light interception and canopy photosynthesis (El-Sharkawy et al. 2012).

To assess the effect of chlorophyll degradation on photoreceptors, Fv/Fm was used as a reliable measure of photoinhibitory impairment in plants under environmental stress, including waterlogging. According to Long et al. (1994), the Fv/Fm ratio is a key parameter for detecting damage to photosystem II (PSII) and identifying the potential occurrence of photoinhibition. During the 15-day waterlogging period in this study, the Fv/Fm data showed nonsignificant differences between treatments, except at 3 DAT (Fig. 3b). This could be attributed to either reversible photoprotective downregulation or irreversible inactivation of PSII during waterlogging. The stability of PSII at 0 and 6–15 DAT suggests that PSII remained unaffected during these periods, likely due to the absence of chlorophyll and carotenoid degradation. In contrast, at 3 DAT, the observed damage to PSII, as indicated by the SPAD values, could be linked to the waterlogging-induced temporary disruption of photosynthetic processes (Fig. 3a). The significant difference at 3DAT may be due to light-induced oxidative stress rather than a direct water stress effect (Navari-Izzo and Rascio 1999) as evident by (Fig. 1b). According to Kaur et al. (2020) review, waterlogging can cause either reversible photoprotective downregulation or irreversible inactivation of PSII, leading to reduced Fv/Fm. This could explain the decrease observed at 3 DAT, with recovery occurring in subsequent measurements. Overall, Fv/Fm data indicate that 15-day waterlogging does not affect chlorophyll degradation or photoreceptors in 3-month-old cassava seedlings. However, the stability of PSII at 6–15 DAT (Fig. 3.b) suggests that PSII recovered immediately the following day when light intensity resumed normality (Fig. 1b), likely due to the absence of chlorophyll and carotenoid degradation indicating lack of serious significant effect of decreased light intensity on 8th (Fig. 1b).

However, the effect of waterlogging on leaf gas exchange showed significant decreases in gs and A in cassava plants at 3 DAT. Specifically, gs, A, and E in WL cassava plants decreased by 41.1%, 7.9%, and 16.1%, respectively, compared to WW plants (Fig. 2b, a, and c). According to Ahmed et al. (2002), the earliest response of plant species to waterlogging is stomatal closure, which limits gas exchange and decreases A and E in many plant species, such as sorghum (Zhang et al. 2019), wheat (Triticum aestivum L.; Zheng et al. 2009), tomato (Solanum lycopersicum L.; Else et al. 1996), and pigeon pea (Cajanus cajan L. Huth; (Bansal and Srivastava 2015). Maai (2024) reported a reduction in gs and SPAD values in finger millet during the early waterlogging stages, resulting in decreased A over the 13-day waterlogging period.

Contrary, Fig. 2a, b & c showed decreasing trends of the same leaf gas exchange parameters A, gs and E even in the well-watered plants as the treatment duration increased. This could be attributed to a slight degree of aging which was observed as the days progressed in the control plant. We believe that a downward trend has been indicated. It was observed because the photosynthetic-related traits were monitored in the same leaf. In the 15-day continuous measurement. In addition, according to Qin et al. (2024) even in the absence of water deficit, prolonged environmental exposure such as high radiation, VPD or heat can change the internal hormonal landscape, for instance Ethylene may promote senescence-like traits or change stomatal sensitivity, leading to a gradual downtrend in gas exchange and accumulation of Abscisic Acid (ABA) in guard cells even without root stress. This can signal partial stomatal closure to preserve leaf water status– a proactive acclimatization rather than stress reaction (Qin et al. 2024). Furthermore, Cytokinin decline is usually observed with age causing reduced photosynthetic enzyme activity and impaired sink strength, ultimately feeding back to reduce A and gs (Qin et al. 2024). Therefore, the decrease in A could be attributed to a reduction in gs, as decreased stomatal conductance under waterlogged conditions is related to reduced root permeability and root hydraulic conductivity under soil anaerobic conditions, as reported by Mielke et al. (2003). Pezeshki (2001) reported similar findings: Reduced stomatal conductance increases internal water stress caused by reduced root hydraulic conductivity. The significant decrease in gs observed in the WL cassava studied indicates stomatal closure, which restricts the channel for water and CO_2_ exchange.

The Ci data showed nonsignificant increases of 6.1%, 1.9%, 4.1%, and 0.8% on 0, 3, 6, and 12 DAT, respectively—and nonsignificant decreases of 11.5% and 21.4% on 9 and 15 DAT—in the WL cassava compared to the WW controls (Fig. 2d). The substantial decrease in gs, with Ci values either unchanged or slightly higher than in WW plants, suggests that stomatal closure does not sufficiently restrict CO_2_ entry into the leaf to lower Ci. This indicates that mesophyll cells were equally well supplied with CO_2_ in WW and WL plants. These results suggest that photosynthetic inhibition under waterlogging was primarily nonstomatal in origin. The positive correlation between Ci and A (Fig. 4a&b) further supports that photosynthetic inhibition occurred despite adequate internal CO₂ levels, establishing nonstomatal limitation as the dominant factor. This could also be due to reduced carboxylation efficiency rather than stomatal limitation (HUANG et al. 2004). A previous study examining self-grafted tomato plants under waterlogging conditions found similar results: Ci increased (3–23%) in both types of tomato plants when compared to the same types of plants under non-waterlogged conditions (Bhatt et al. 2015). Overall, reduced photosynthesis could be due to nonstomatal limitations.

However, the leaf gas exchange measurements indicated possible stomatal limitations for transpiration and photosynthesis in the WL plant leaves (Fig. 2a, b, and c). Significant increases in PWUE were observed in the WL plants compared to the WW plants at 6 and 15 DAT (Fig. 2e). This is due to the reduced root hydraulic conductivity, which increases internal water stress, leading to decreased leaf turgor and stomatal conductance in WL plants (Davies and Flore 1986; Pezeshki 2001). In addition, this difference suggests that waterlogging affects the nonstomatal component of photosynthesis (Maai 2024). However, a significant difference existed between the treatment durations (Fig. 2e).

Furthermore, the Pearson correlation matrix performed for both WW and WL treatments results showed a positive correlation between A and gs, and between E and gs (Fig. 4a & b). The relation between stomata conductivity and transpiration in Fig. 4a seems to be evident. These data suggest that the reduction in gs triggers a decrease in A and E during the early waterlogging stage. Therefore, stomatal closure should be considered an avoidance mechanism that enhances cassava survival under waterlogged conditions. This is in agreement with El-Sharkawy et al. (2012) who reported that cassava conserves water and prevents extreme leaf dehydration through stomatal sensitivity to stress- uses it as a stress avoidance mechanisms.

In waterlogging, the gas exchange function of the leaves is also reduced due to lack of oxygen in the roots, but both stomatal and non-stomatal factors are involved, and non-stomatal factors, such as a decrease in the amount of electron transfer chains and chlorophyll, may be the main limiting factors, but non-stomatal damage may be better explained by using Fv/Fm as an indicator. So, the reduced net photosynthetic rate observed with increasing waterlogging duration and between the treatments could be due to stomata and nonstomatal limitations. While the relationship between photosynthesis and SPAD value as follows. From the results of this study, it is inferred that the reaction of the effect of the decrease in photosynthetic capacity on the SPAD value was slow, and that the decrease in the SPAD value was not significant during the experimental period.

## Conclusion

The Tokunoshima Yellow 2 cassava cultivar was subjected to 15-day waterlogging stress to investigate its physiological responses. The study analyzed responses related to photosynthesis, relative chlorophyll content, stomatal conductance, and Fv/Fm in leaves, aiming to determine chlorophyll breakdown and its effect on photoreceptors. The effect of waterlogging on photosynthesis varied with duration. No severe effects were observed on SPAD value and Fv/Fm, indicating no degradation of chlorophyll content or impact on photoreceptors, as well as no inhibition of PSII. Thus, the lack of a nonsignificant difference in SPAD indicates that the early reduction in the net photosynthetic rate in WL cassava plants may result from stomatal conductance limitation. The initial decreases in gs, E, and A caused by waterlogging indicate the restriction of the water and CO₂ exchange channels. However, data on Ci values, which were either unchanged or slightly higher than those in the WW plants, imply that stomatal closure did not restrict CO₂ entry into the leaf enough to reduce Ci. This suggests that mesophyll cells were equally well supplied with CO₂ in WW and WL plants. As a result, reductions in photosynthesis could be attributed mostly to nonstomatal limitations. This could also result from reduced carboxylation efficiency rather than stomatal limitations. Overall, the reduced net photosynthetic rate observed with increasing waterlogging duration and between the treatments could be due to stomata and nonstomatal limitations. It was also observed that Cassava exhibits a functional stay-green type of SPAD, and Photosynthetic Nitrogen use efficiency that regulate photosynthesis under waterlogged conditions. However, more studies are needed to confirm these findings. This study establishes understanding of the physiological adaptations which provide valuable insights into how cassava cope with waterlogging stress and guide breeding or agronomic strategies to improve their resilience in waterlogged environments. This approach may involve exploiting genetic variations in leaf anatomy and biochemistry to enhance photosynthetic efficiency and crop productivity as suggested by (El-Sharkawy 1990).

## Data Availability

Data will be made available on request.
